# Supplementary material for: Different hotspot p53 mutants exert distinct phenotypes and predict outcome of colorectal cancer patients
Source: Nat Commun. 2022 May 19;13:2800. doi: 10.1038/s41467-022-30481-7 (PMC9120190; doi:10.1038/s41467-022-30481-7)
Supplement: Supplementary file 1 — Supplementary Information [file 41467_2022_30481_MOESM1_ESM.pdf]

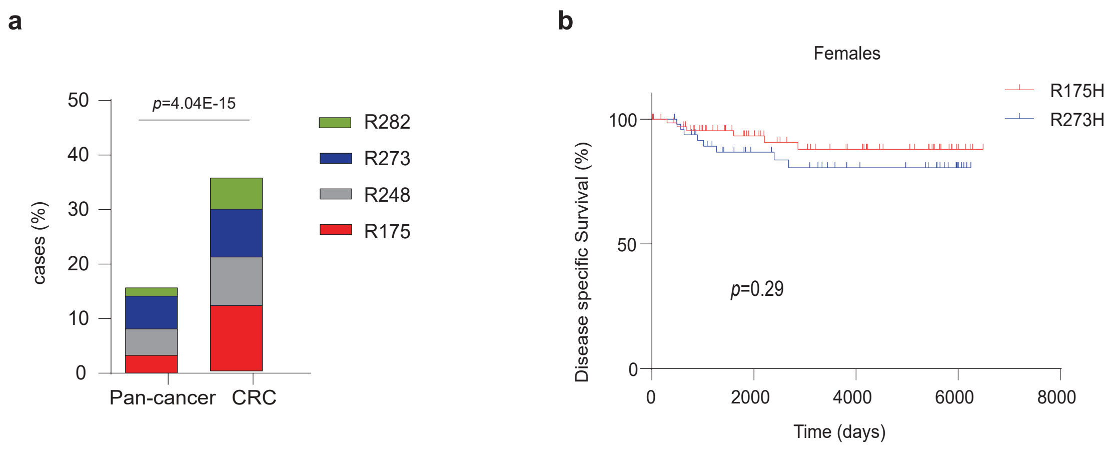

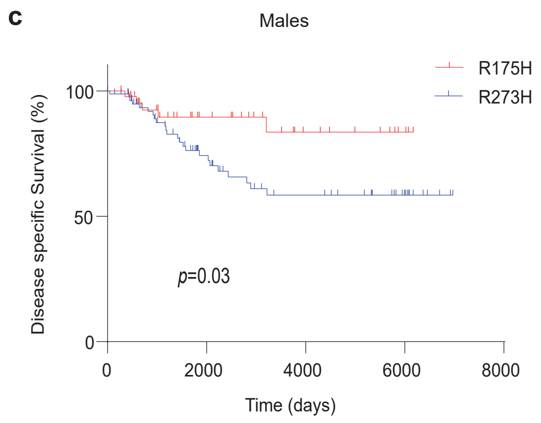

d

	APC	KRAS	SMAD4	BRAF	PIK3CA	FBXW7	PTEN
R175	67.0%	28.3%	10.2%	7.8%	13.1%	13.8%	4.6%
R273	63.1%	32.4%	16.5%	12.5%	15.8%	12.8%	5.7%
P-value	0.33	0.32	0.02*	0.07	0.40	0.80	0.57

Supplementary Fig. 1. TP53 R273 mutations are associated with more aggressive colorectal tumors and shorter overall survival relative to R175 mutations.

a, Relative abundance of mutations in the top four TP53 hotspot mutational sites in colorectal cancer (CRC, n=323) versus all other cancers (Pan-cancer, n=3396) in TCGA. Shown is the % of cases with mutations in each of the indicated hotspot residues out of all TP53-mutated cases. ****P-value <0.0001 (Two sided Fisher's exact test). b-c, Disease specific survival of CRC patients with either R175 or R273 mutations sorted by gender. Compiled from TCGA COAD-READ and published data (24). Log-rank test. d, Co-occurrence of CRC tumors harboring either R175 (N=282) or R273 (N=296) mutations with different driver mutations. For each gene, the percentage describes the number of CRC tumors harboring mutations in both genes together, out of the total number of tumors with TP53 mutations at the indicated residue (R175 or R273). Statistical significance for co-occurrence was calculated using Fisher's exact test. *P-value <0.05.

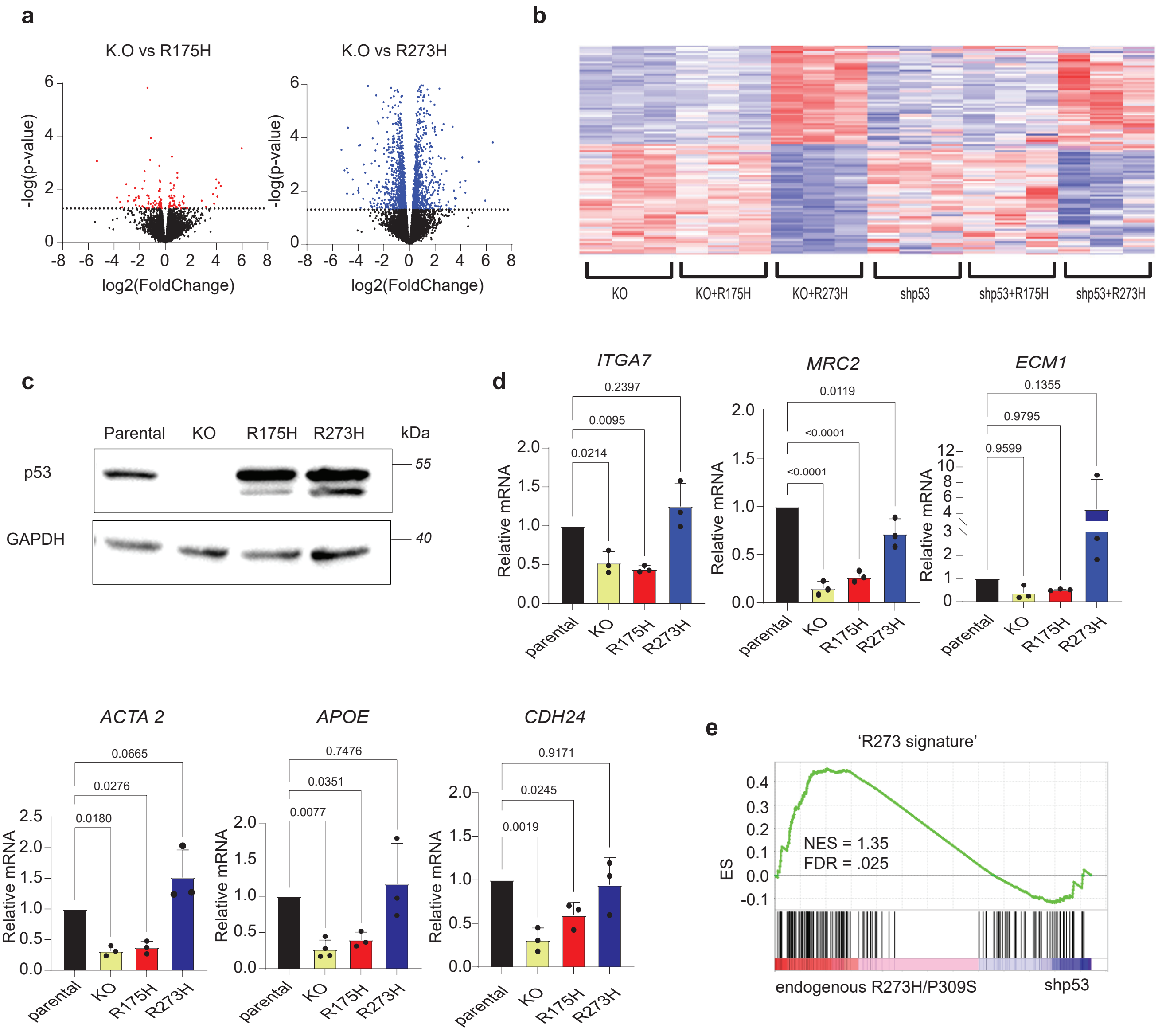

Supplementary Fig. 2. p53R273H orchestrates a distinct transcriptional signature

a, Volcano plots depicting the relative expression of individual genes in SW480 p53 KO cells, compared to their derivatives stably overexpressing either p53R175H (left) or p53R273H (right), deduced from the RNA-seq analysis. Each dot represents a gene. Dashed line represents a cutoff of p-value <0.05. b, Overlay of heatmaps of the differentially expressed genes (fold change>1.5, Padj<0.05) between SW480 p53 KO and their derivatives overexpressing either p53R175H or p53R273H (left 3 columns), and of the same genes in SW480 cells stably expressing p53 shRNA (shp53) without or with stable overexpression of shRNA-resistant p53R175H or p53R273H (right 3 columns), deduced from RNA-seq (n=3 for each condition). c, Western blot analysis of p53 protein in parental SW480 cells, SW480 cells after CRISPR/Cas9 p53 knockout (KO), and p53 KO cells stably overexpressing p53R175H or p53R273H. d, RT-qPCR analysis of representative R273 signature genes in parental SW480 cells, SW480 p53 KO cells, and p53 KO cells stably overexpressing p53R175H or p53R273H. Mean + SEM of Three biological repeats. *P-value<0.05; **P-value<0.01, One-way ANOVA and Tukey's post hoc test.. e, GSEA enrichment plot for SW480 RNA-seq data ranked by fold change upon mutp53 depletion (33), using the R273H signature as the tested gene set. ES = enrichment score.

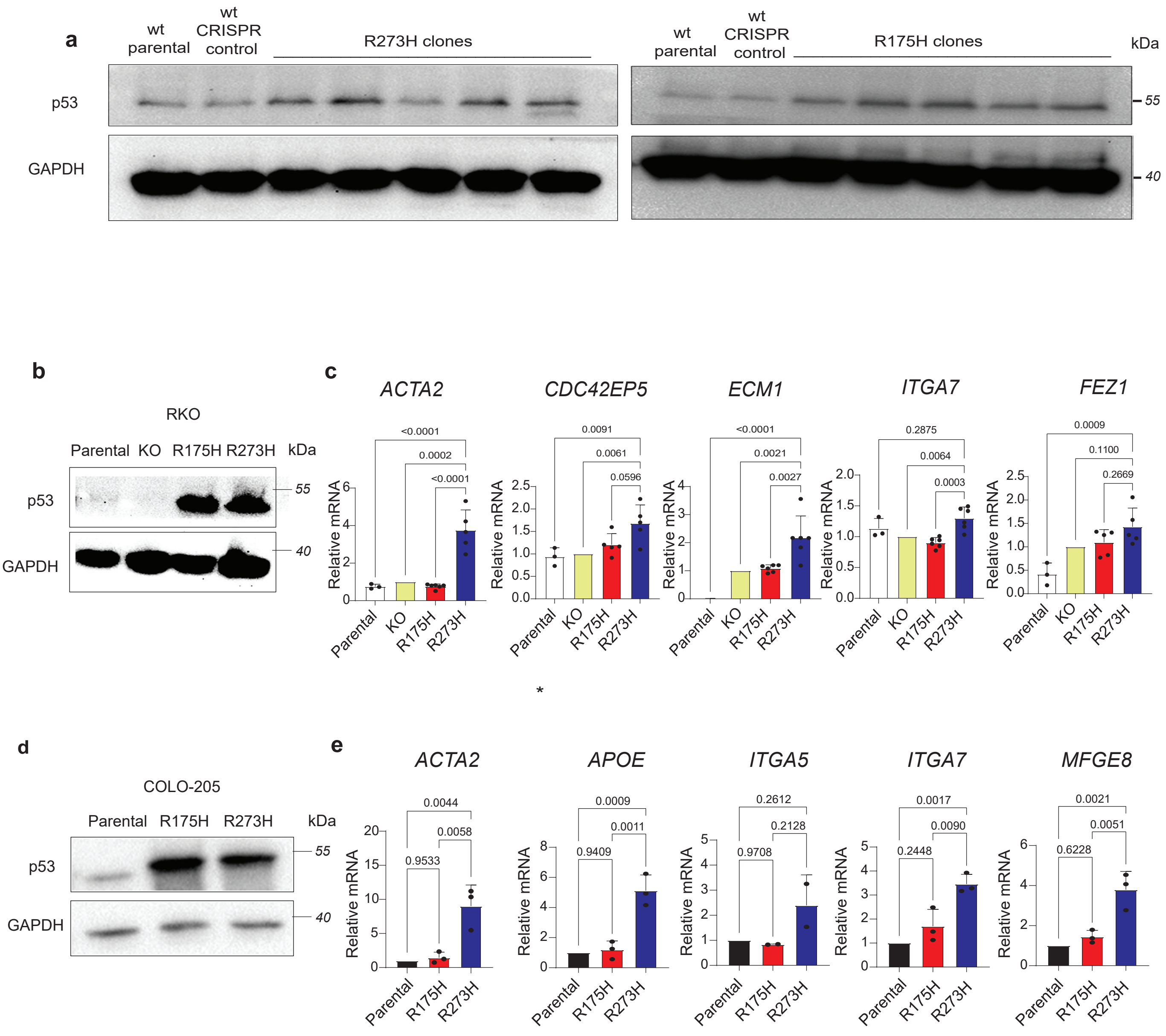

Supplementary Fig. 3. The R273 signature is upregulated in CRC cell lines and tumors

a, Western blot analysis of p53 protein levels in 5 individual HCT116-derived clones from each indicated CRISPR/Cas9 mutp53 knock-in, compared to parental wtp53-expressing HCT116 cells (wt parental) and a wtp53 CRISPR/Cas9 control clone (wt CRISPR), n=2. b, Western blot analysis of parental RKO cells, p53 knockout (KO) RKO cells and p53 KO derivatives stably overexpressing either p53R175H or p53R273H. c, RT-qPCR analysis of the expression of representative R273 signature genes in the cells in (b). Values were normalized to GAPDH mRNA and are shown relative to the control KO cells. Mean + SEM from at least three independent repeats (one-way ANOVA and Tukey's post hoc test). d, Western blot analysis of p53 in parental COLO-205 cells and their derivatives stably overexpressing either p53R175H or p53R273H. e, RT-qPCR analysis of representative R273 signature genes in the cells in (d), Mean + SEM from three independent biological repeats. One-way ANOVA and Tukey's post hoc test.

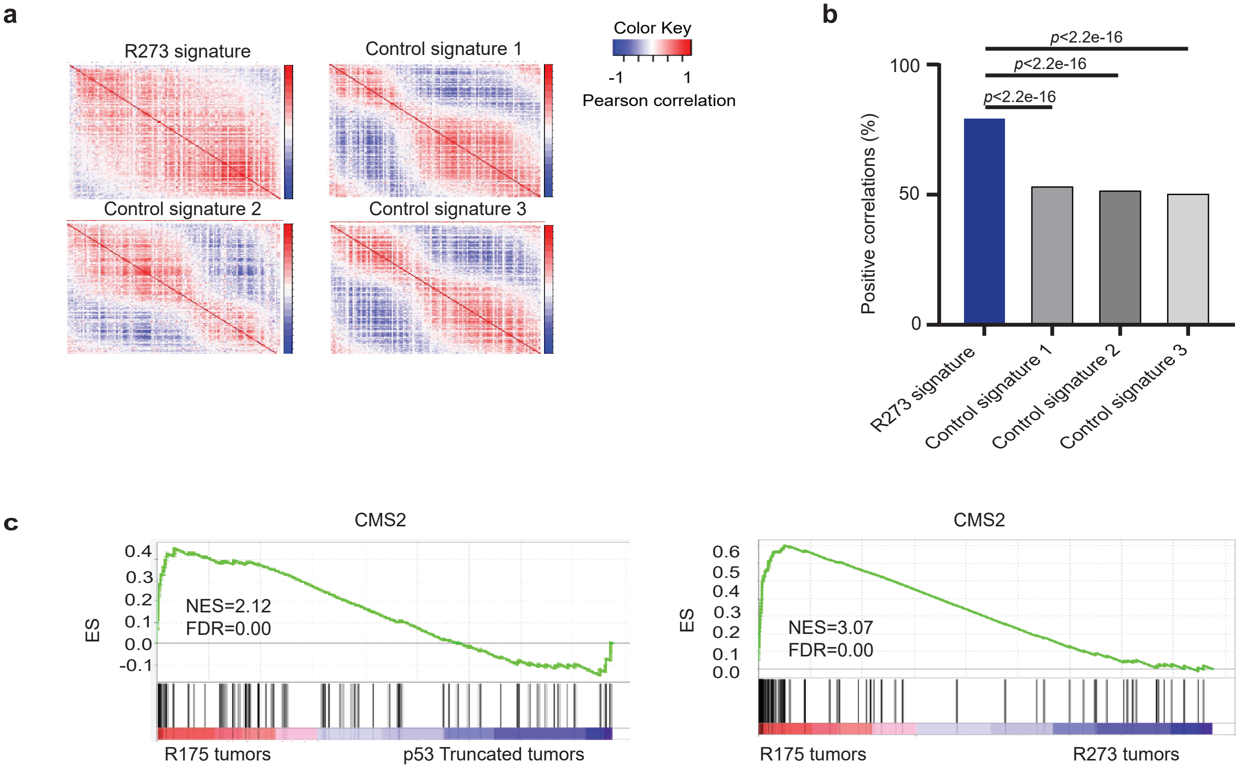

Supplementary Fig. 4. R273 signature genes are co-expressed in CRC while R175-mutated CRC is associated with the CMS2 gene signature.

a, Correlogram of the genes in the R273 signature and in three independent control signatures in the TCGA colorectal cancer cohort (n=635), demonstrating extensive co-expression of the R273 signature genes. Correlation was calculated by Pearson coefficient (see expression ladder on right). Each control signature comprised an equal number of genes as the R273 signature, having an adjusted P-value>0.98 (non-differentially expressed genes) in our RNA-seq. b, Percentage of positive correlations out of the total correlations (N=18,496 correlations for R273 signature and 18,225 or 17,956 for control signatures) in the R273 signature and in the control signatures in (a). Pearson correlation coefficient was used to calculate correlations between genes, and two sided Fisher's exact test was used to calculate statistical differences between groups. c, GSEA of CRC tumors harboring R175 mutations (n=36) compared to tumors harboring truncating mutations (n=28; left) or to tumors harboring R273 mutations (n=28; right). The CMS2 gene signature was used as the tested gene set.

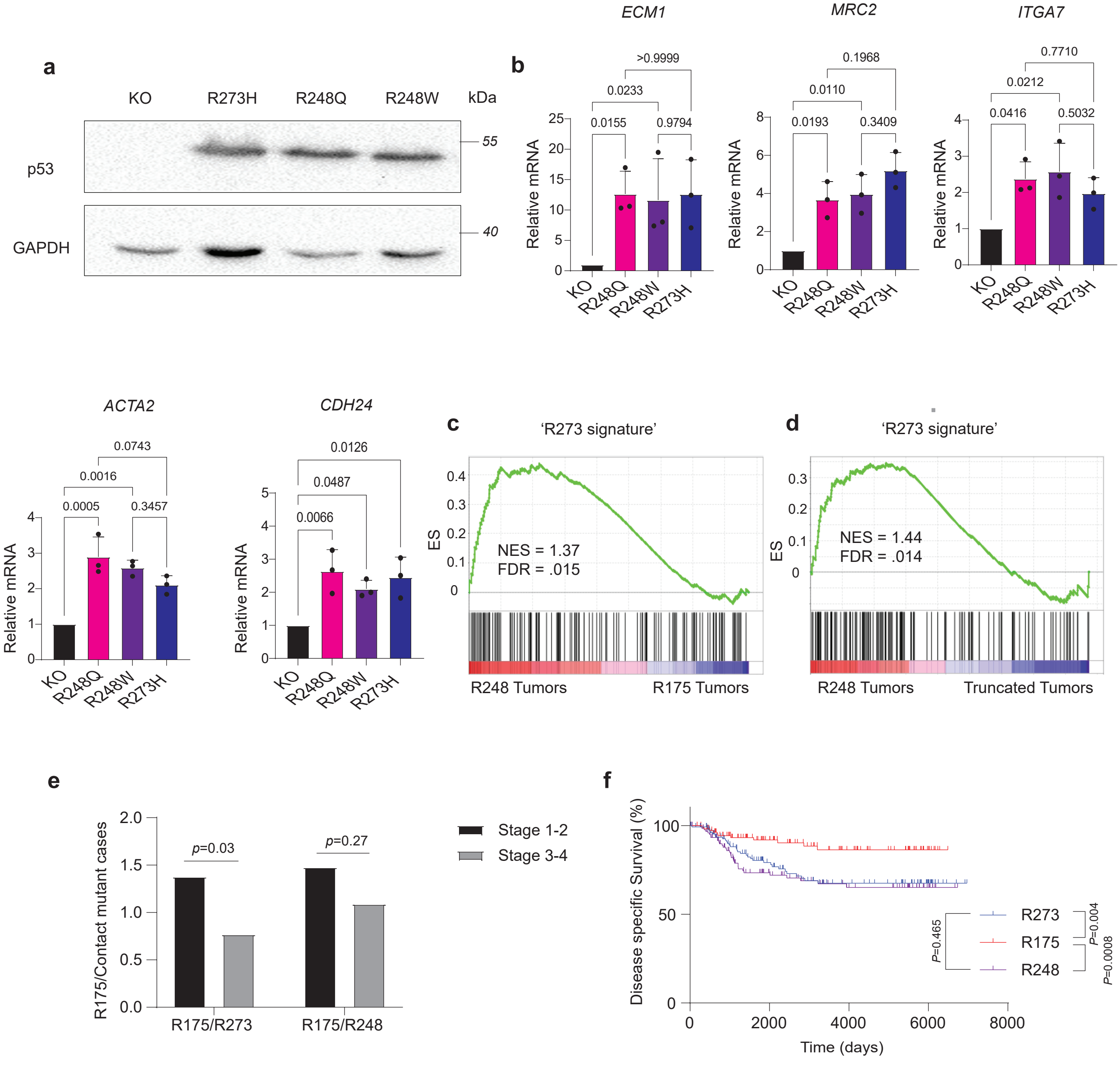

Supplementary Fig. 5. R248 TP53 hotspot mutants in CRC are associated with the R273 signature and with reduced survival relative to R175 mutants.

a, Western blot analysis of p53 in SW480 knockout (KO) cells without or with stable overexpression of the indicated TP53 hotspot mutants. b, RT-qPCR analysis of representative R273 signature genes in the cells depicted in (a). Three biological repeats. One-way ANOVA and Tukey's post hoc test. c-d, GSEA of CRC tumors harboring R248 mutations (n=30) compared to tumors harboring R175 (n=36) or truncating (n=28) mutations; truncating mutations were the same as in Fig. 4b. Genes were ranked by fold change, and the R273 signature was used as the tested gene set. e, Ratio between the numbers of CRC cases with R175 mutations (N=132) vs cases with R273 (N=121) or R248 mutations (N=100) in stage 1-2 and in stage 3-4 disease. The R273 data (left) is the same as in Fig. 1b, and is provided for comparison. Two sided Fisher's exact test. f, Disease specific survival of CRC patients with either R175, R248 or R273 mutations. Compiled from TCGA COAD-READ and published data (24). Log-rank test. The R175 and R273 data is the same as in Fig. 1e, and is provided for comparison.

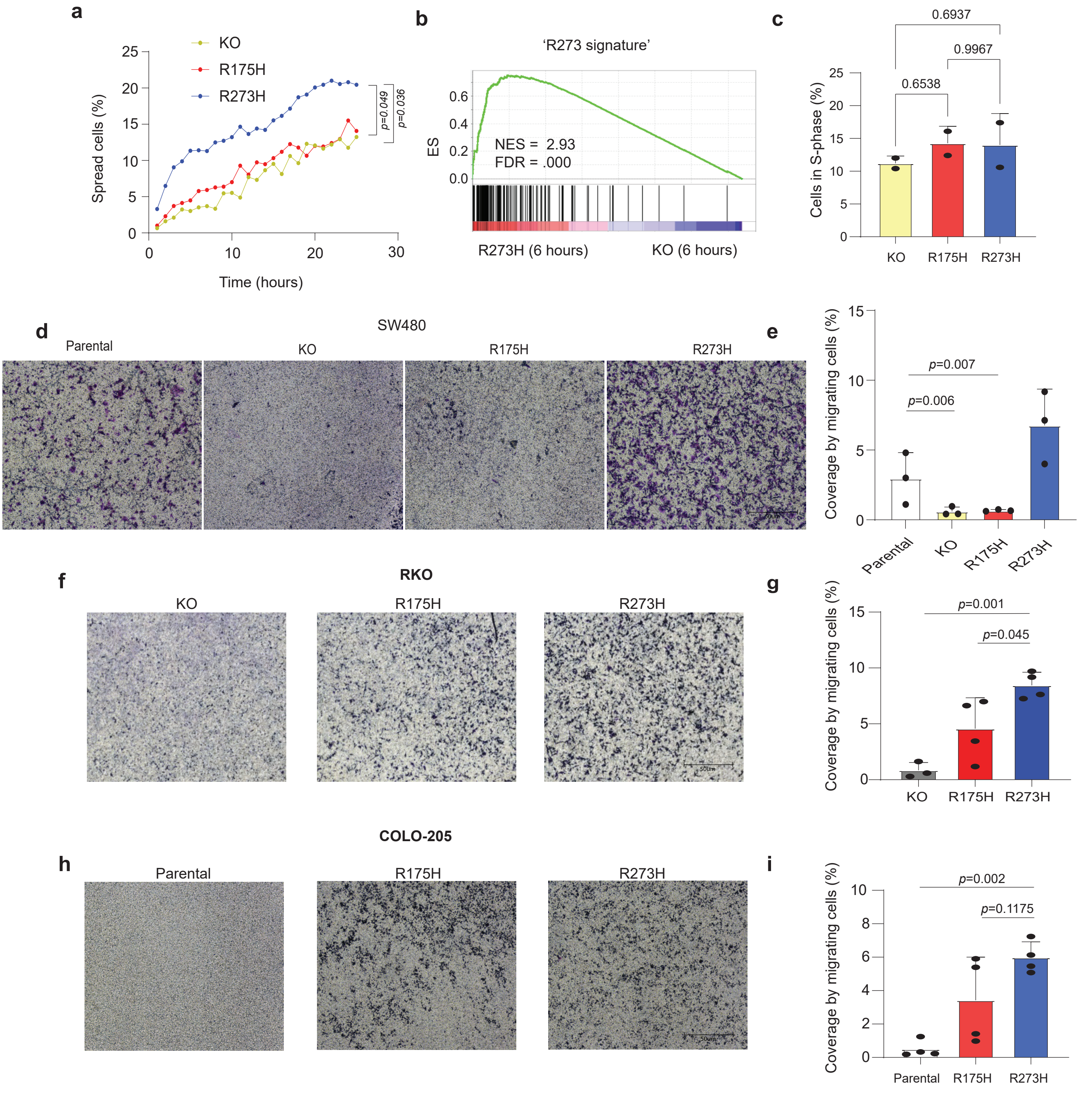

Supplementary Fig. 6. p53R273H promotes cell spreading, migration and invasion.

a, Kinetics of spreading of RKO p53 KO cells (KO) and their derivatives stably overexpressing p53R175H or p53R273H. Statistical analysis at t=23 was done using one-way ANOVA and Tukey's post hoc test, n=2. b, GSEA plot of the transcriptomes of SW480 KO cells and their derivatives stably overexpressing p53R273H, six hours post seeding. Genes was ranked by fold change upon p53R273H overexpression and the R273H signature was used as the tested gene set. c, SW480 TP53 KO cells and their derivatives stably overexpressing p53R175H or p53R273H were subjected to cell cycle profiling by BrdU and DAPI staining. The bar graph represents the average percentage of cells in S phase from two biological repeats (Mean + SEM). One-way ANOVA and Tukey's post hoc test. d, Representative images of transwell migration assays performed with parental SW480 cells, SW480 p53 KO cells, and p53 KO derivatives stably overexpressing p53R175H or p53R273H. e, Average percentage of area coverage (ImageJ) by migrating cells in transwell migration assays as described in (d). Mean + SEM of three biological repeats. One-way ANOVA and Tukey's post hoc test. f, Representative images of transwell migration assays performed with RKO p53 KO cells and their derivatives stably overexpressing p53R175H or p53R273H. g, Average percentage of area coverage (ImageJ) by migrating cells in transwell migration assays as described in (f). Mean + SEM of two biological repeats. One-way ANOVA and Tukey's post hoc test. h, Representative images of transwell migration assays performed with parental COLO-205 cells and their derivatives stably overexpressing p53R175H or p53R273H. i, Average percentage of area coverage (ImageJ) by migrating cells in transwell migration assays as described in (h). Mean + SEM of four biological repeats. One-way ANOVA and Tukey's post hoc test.

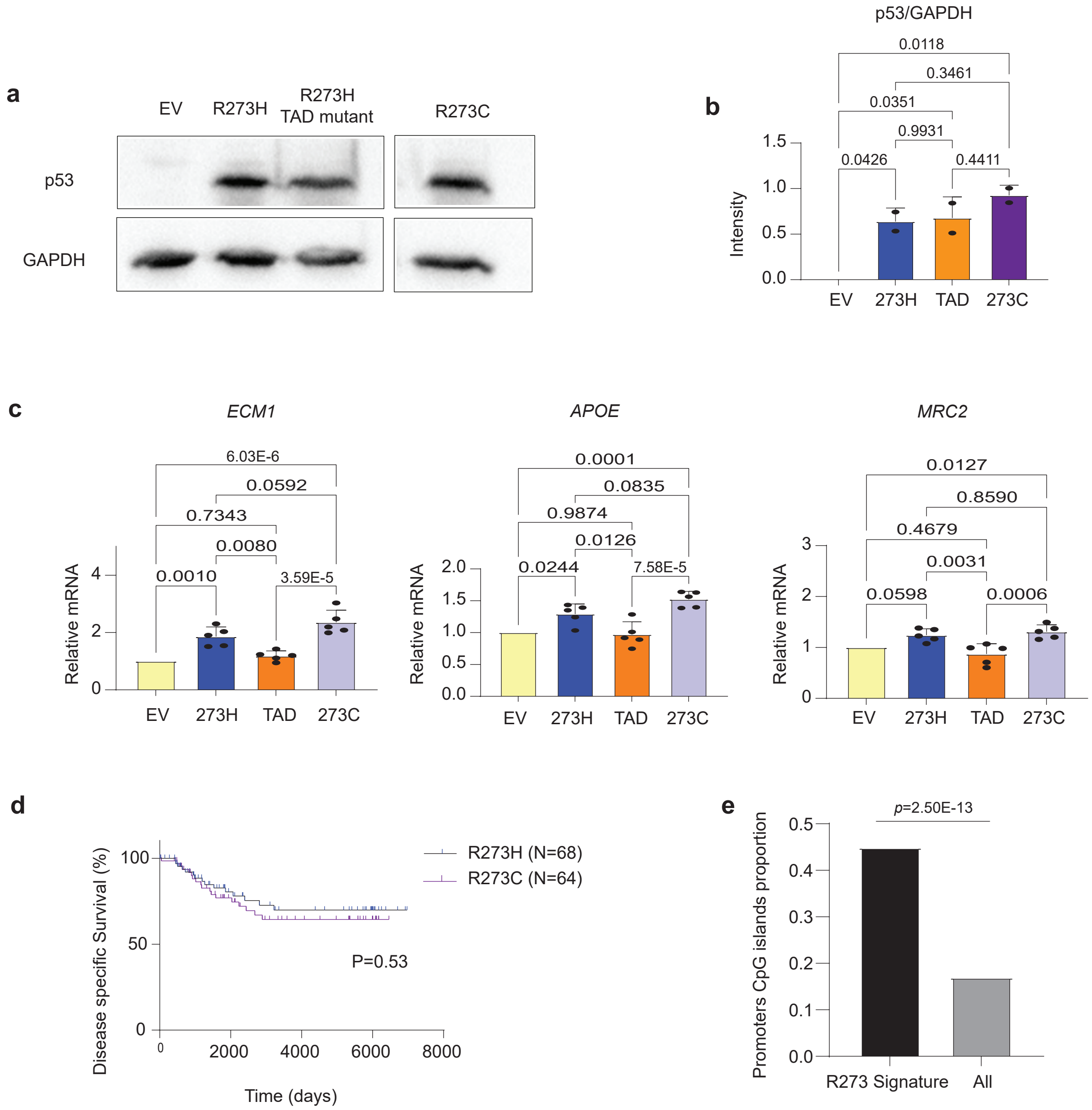

Supplementary Fig. 7. Regulation of R273 signature genes by p53R273H and p53R273C.

a, Western blot analysis of p53 in SW480 p53 KO cells, harvested 48 hours after transient transfection with empty vector (EV) or with DNA encoding either p53R273C, p53R273H or p53R273H carrying a double mutation in residues 22,23 within the transactivation domain (R273H TAD mutant). b, Quantification of Western blot data as in (a), from two biological repeats, using Image Lab (Bio-Rad). “TAD” refers to the 273H TAD mutant. One-way ANOVA and Tukey's post hoc test of the indicated comparisons. Two biological repeats. c,RT-qPCR analysis of representative R273 signature genes in SW480 p53 KO cells transiently transfected as in (a), one-way ANOVA and Tukey's post hoc test of five biological repeats. d, Disease specific survival of CRC patients with either R273C or R273H mutations. Compiled from TCGA COAD-READ and published data (24). Log-rank test. e, Proportion of CpG islands in canonical promoters of the R273 gene signature vs proportion of CpG islands in all human genome promoters. CpG islands in promoters of the R273 gene signature (N=57) were compared to all canonical gene promoters (N=59,197) as described in Methods under Transcription factor enrichment. Calculation of overlap between CpG islands and promoters was done using bedtools intersect (version 2.25.0) and a minimum of 1 bp overlap between CpG Island and the promoter was considered positive. Chi square analysis was used as the statistical test.

Supplementary Table 1: Univariate and multivariate analyses of overall survival in CRC tumors with mutations in R175 vs mutations in R273 (Cox proportional regression model)

	**Univariate analysis**			**Multivariate analysis**		
**Factor**	HR	95% CI	*p* -value	HR	95% CI	*p* -value
R273/R175 mutation	2.596	1.309-5.152	0.0046*	2.291	1.133-4.631	0.02*
Male/Female	2.28	1.204-4.318	0.009*	1.958	1.022-3.751	0.042*
Age at diagnosis	1.004	0.975-1.-035	0.8	1.009	0.978-1.041	0.571
KRAS mutation Yes/No	1.302	0.694-2.439	0.4	1.203	0.638-2.262	0.568
Rectal/colon	0.994	0.501-1.974	1	0.953	0.477-1.905	0.893

Supplementary Table 2: Genes comprising the R273 signature

EEF1A2	TMEM173	CDC42EP1	CERCAM
HES7	GPR162	TP53I11	SMG6
MRC2	CPNE2	ABHD8	MARK4
CHD3	QPRT	GRIN2D	SELENOM
CCDC69	MFGE8	RILP	RAC2
PTPN7	HLA-DQB1	PPP1R1A	FAM171A2
CAMK1	H1FX	KRT17	ITGA5
CDC42EP5	LAT2	C1orf61	COL13A1
MEIS3	TM7SF2	CD99L2	SLC41A1
NRGN	CD4	IL17RE	DEF6
FXR2	REEP2	AC104581.1	PCDH1
H1F0	SMPDL3B	TNNI3	DGKA
CHST1	C9orf16	APBB1	TPI1P1
CAPN5	ACTA2	SBK1	SHISA4
HIST1H1C	MVP	LIMK1	DNM1
APOE	MYRF	CDH24	ADCY6
CD52	P2RX5	STARD10	CHPF2
ITGA7	CAMTA2	DLG4	PROCR
DLK2	SELENBP1	TSPAN14	FBLIM1
HLA-B	TAX1BP3	SHC1	SSBP3
CLEC11A	CX3CL1	SCD5	LRRC8E
HCP5	VAMP5	RIMS4	RASGRF1
ZBTB4	IGFBP7	CD70	LIMK2
ITM2C	HAGLROS	HMGN2	NXPH4
SULT1A1	ECM1	SNCG	SRC
SMARCD3	CXCL16	C19orf54	NATD1
FXYD6	TNFRSF10D	NLGN2	HIST1H2BD
CACNB3	SHF	EVA1B	LMO1
ASRGL1	CLSTN3	PTMS	ZNF219
GYS1	PLEKHA4	MST1	NEURL1B
EGR1	PCBP4	B3GAT1	FEZ1
POLR2A	RTN1	SLC22A17	PEA15
BEX4	GCHFR	NECAB3	KCTD11
PRRX2	ARC	TMEM132A	BORCS6
NTN1	INPP5K	LRP1	WDR45

Supplementary Table 3: R273 signature genes differentially expressed in R273 mutant CRC tumors, relative to both R175 mutant CRC tumors and CRC tumors with truncating p53 mutations. Only the genes that contributed most to the GSEA enrichment result (leading-edge subset, as defined by the GSEA website) are included in the Table.

PROBE	
HLA-B	BEX4
C1orf61	FAM171A2
DEF6	SHF
FEZ1	TNNI3
HCP5	NLGN2
CD70	APBB1
VAMP5	CD52
ECM1	ITGA5
MFGE8	RAC2
B3GAT1	GPR162
SLC22A17	SHISA4
MRC2	SCD5
CERCAM	SMARCD3
NTN1	ITGA7
MEIS3	DNM1
DLG4	PRRX2
HLA-DQB1	RTN1
SLC41A1	LAT2
CLSTN3	CCDC69
PPP1R1A	APOE
EEF1A2	REEP2

Supplementary Table 4: Univariate and multivariate analyses of overall survival in CRC tumors with high R273 signature vs low R273 signature (Cox proportional regression model)

	Univariate analysis			Multivariate analysis		
**Factor**	HR	95% CI	*p* -value	HR	95% CI	*p* -value
h signature/Low signature express	2.169	1.276-3.687	0.003*	2.314	1.344-3.977	0.002*
Male/Female	0.988	0.593-1.645	0.964	1.067	0.631-1.803	0.807
Age at diagnosis	0.98	0.996-1.044	0.1	1.022	0.998-1.047	0.06
KRAS mutation Yes/No	0.677	0.391-1.174	0.2	0.64	0.367-1.114	0.11
Rectal/colon	0.653	0.330-1.291	0.2	0.658	0.330-1.310	0.234

Supplementary Table 5: gRNA and ssODN sequences

R175H gRNA and ssODN

GCAGTCACAGCACATGACGG

GGCAACCAGCCCTGTCGTCTCTCCAGCCCCAGCTGCTCACCATCGCTATCTGAGCAGCGCTCATGGTGGGGGCAGtGCCTCACAACtTCCGTCATGTGCTGTGACTGCTTGTAGATGGCCATGGCGC

R273H gRNA and ssODN

ACTGGGACGGAACAGCTTTG

tttccttactgccTCTTGCTTCTCTTTTCCTATCCTGAGTAGtggtaatctactgggacgcaacagttttgaagtgcatgtttgtgcctgtcctgggagagaccggcgcacagaggaagagaatctccgcaagaaagg

Supplementary Table 6: List of primers used in this study

**Primers for RT-qPCR**	**Sequence**
GAPDH _fw	GGAAGGTGAAGGTCGGAGTC
GAFDH _rv	TGAGGTCAATGAAGGGGTCA
TP53 _fw	CCAAGCAATGGATGATTTGA
TP53 _rv	GGCATTCTGGGAGCTTCATCT
ACTA2 _fw	AAGATCCTGACTGAGCGTGG
ACTA2 _rv	GCAGTGGCCATCTCATTTTC
ECM1 _fw	ACACAAACCGCCTAGAGTGTG
ECM1 _rv	AACACAAGTGGTGGTGGGTC
APOE _fw	GGAAGATGAAGGTTCTGTGGG
APOE _rv	TCTGCCACTCGGTCTGCT
CDC42EP5 _fw	CGAGAGGGGCTGACACTTTA
CDC42EP5 _rv	CTCCGTCTCCTAATCCCCG
MRC2 _fw	ATGTGTGCAAGAAGAAGCCC
MRC2 _rv	CTCGCACTCCACCTTCACAT
ITGA7 _fw	GTTGGGAGTCAGTGTTCGGA
ITGA7 _rv	AGCAGCGACCAATCATATCC
FEZ1 _fw	AAACTTCCTCCCAGGCAGAC
FEZ1 _rv	GGTCAGCTCAGACCCAGACA
CDH24 _fw	CTGGTAGGCCAGATCTCCG
CDH24 _rv	CCTCGGGCTGGATAGAGAAG
MFGE8 _fw	CGGGCAACCACTGTGAGAC
MFGE8 _rv	AATGCTGCAAACCCAAGAAG
ITGA5 _fw	CAGGGTGGTGCTGTCACCT
ITGA5 _rv	GCTCAGTGGCTCCTTCTCTG
PLEKH4 _fw	CCCACACACTGAGCCTGAC
PLEKH4 _rv	CTAGGGGAGCTCAGTTCCAAG
EGR1 _fw	CTTCAACCCTCAGGCGGACA
EGR1 _rv	GGAAAAGCGGCCAGTATAGGT
**Primers for CHIP**	Sequence
ITGA7 _Regulatory element_fw	AAGGAGCGACTCTGGAGGGA
ITGA7 _Regulatory element_rv	GTGGTTTCAGGCCTCCAAGAAG
APOE _Regulatory element_fw	CGTTGCTGGTCACATTCCTGG
APOE _Regulatory element_rv	GCAGCACAGAAGCCTCAGAAG
ITGA7 _Intronic_fw	CTCCTTCTGCCTCCAATGCT
ITGA7 _Intronic_rv	AGAGGCGTTGAGGTTTGTCC
APOE _Intronic_fw	AATGCATTGCAGGCAGATAGTG
APOE _Intronic_rv	GTGTGGGGGTGATGGAGAATA
