# Supplementary material for: Different hotspot p53 mutants exert distinct phenotypes and predict outcome of colorectal cancer patients
Source: Nat Commun. 2022 May 19;13:2800. doi: 10.1038/s41467-022-30481-7 (PMC9120190; doi:10.1038/s41467-022-30481-7)
Supplement: Supplementary file 3 — Description of Additional Supplementary Files [file 41467_2022_30481_MOESM3_ESM.pdf]

Description of Additional Supplementary Files

File Name: Supplementary Data 1
Description: CRC tumors harboring R175 or R273 mutations stratified by tumor stage. Data extracted from TCGA and ICGC open-source platforms as well as from additional published datasets (22,23,24).

File Name: Supplementary Movie 1
Description: SW480 KO cells (a) were subjected to time lapse microscopy. Red arrows indicate cells undergoing cell spreading in the course of the 24 hours of the experiment.

File Name: Supplementary Movie 2
Description: SW480 R175H cells (b) were subjected to time lapse microscopy. Red arrows indicate cells undergoing cell spreading in the course of the 24 hours of the experiment.

File Name: Supplementary Movie 3
Description: SW480 R273H cells (c) were subjected to time lapse microscopy. Red arrows indicate cells undergoing cell spreading in the course of the 24 hours of the experiment.
