# Supplementary material for: Different hotspot p53 mutants exert distinct phenotypes and predict outcome of colorectal cancer patients
Source: Nat Commun. 2022 May 19;13:2800. doi: 10.1038/s41467-022-30481-7 (PMC9120190; doi:10.1038/s41467-022-30481-7)
Supplement: Supplementary file 8 — Reporting Summary [file 41467_2022_30481_MOESM8_ESM.pdf]

nature portfolio

Corresponding author(s): Moshe Oren

Last updated by author(s): 24/3/22

Reporting Summary

Nature Portfolio wishes to improve the reproducibility of the work that we publish. This form provides structure for consistency and transparency in reporting. For further information on Nature Portfolio policies, see our Editorial Policies and the Editorial Policy Checklist.

Statistics

For all statistical analyses, confirm that the following items are present in the figure legend, table legend, main text, or Methods section.

n/a	Confirmed	
☐	☒	The exact sample size (*n*) for each experimental group/condition, given as a discrete number and unit of measurement
☐	☒	A statement on whether measurements were taken from distinct samples or whether the same sample was measured repeatedly
☐	☒	The statistical test(s) used AND whether they are one- or two-sided<br>*Only common tests should be described solely by name; describe more complex techniques in the Methods section.*
☒	☐	A description of all covariates tested
☒	☐	A description of any assumptions or corrections, such as tests of normality and adjustment for multiple comparisons
☐	☒	A full description of the statistical parameters including central tendency (e.g. means) or other basic estimates (e.g. regression coefficient) AND variation (e.g. standard deviation) or associated estimates of uncertainty (e.g. confidence intervals)
☐	☒	For null hypothesis testing, the test statistic (e.g. *F*, *t*, *r*) with confidence intervals, effect sizes, degrees of freedom and *P* value noted<br>*Give P values as exact values whenever suitable.*
☒	☐	For Bayesian analysis, information on the choice of priors and Markov chain Monte Carlo settings
☒	☐	For hierarchical and complex designs, identification of the appropriate level for tests and full reporting of outcomes
☐	☒	Estimates of effect sizes (e.g. Cohen's *d*, Pearson's *r*), indicating how they were calculated

Our web collection on statistics for biologists contains articles on many of the points above.

Software and code

Policy information about availability of computer code

Data collection	TCGA data was collected from UCSC-xena or cBioportal.
Data analysis	Partek Genomics Suite (Partek Inc, v7.0), ImageJ (v1.53c), ilastik (v1.3.3), Image Lab (Bio-Rad, v4.1), Graph-Pad Prism (v9.1.0), Celldiscoverer 7 microscope (Carl Zeiss Ltd.), UTAP pipline (v1.0.9), Snakemake (v1.0.68), GSEA (v4.0.0), bedtools intersect (v2.25.0), GGA (v20210531), MatBase (v11.3). FastQC (v0.11.9), DESeq2 (v1.30.1), BETA (v1.0.7)

For manuscripts utilizing custom algorithms or software that are central to the research but not yet described in published literature, software must be made available to editors and reviewers. We strongly encourage code deposition in a community repository (e.g. GitHub). See the Nature Portfolio guidelines for submitting code & software for further information.

Data

Policy information about availability of data

All manuscripts must include a data availability statement. This statement should provide the following information, where applicable:

- Accession codes, unique identifiers, or web links for publicly available datasets
- A description of any restrictions on data availability
- For clinical datasets or third party data, please ensure that the statement adheres to our policy

All sequencing data that support the findings of this study have been deposited in the National Center for Biotechnology Information Gene Expression Omnibus (GEO) and are accessible through GEO Series Accession Number GSE173364 (https://www.ncbi.nlm.nih.gov/geo/query/acc.cgi?acc=GSE173364).
All other relevant data are available from the corresponding author upon reasonable request
Public ChIP-seq data was downloaded from the SRA database (accessions: SRR5944061, SRR5944062, SRR5944081)

Field-specific reporting

Please select the one below that is the best fit for your research. If you are not sure, read the appropriate sections before making your selection.

☒ Life sciences ☐ Behavioural & social sciences ☐ Ecological, evolutionary & environmental sciences

For a reference copy of the document with all sections, see nature.com/documents/nr-reporting-summary-flat.pdf

Life sciences study design

All studies must disclose on these points even when the disclosure is negative.

Sample size	No sample size calculations were performed. Animal sample size was determined by the literature and the number of biological replicates necessary for ensuring statistical significance. The number of biological replicates are reported in the relevant figure legends in the manuscript.
Data exclusions	No data was excluded
Replication	Experiments were performed at least 2 times independently. Similar observation was obtained for each replicate. Representative result was shown in the figures
Randomization	No randomization was performed. Most comparisons were performed between determined status
Blinding	The experiments and analysis were performed in a non-blinded fashion because the same investigator was doing group allocation during data collection and analysis. Our analyses all generate objective outcomes that are not subject to observer bias.

Reporting for specific materials, systems and methods

We require information from authors about some types of materials, experimental systems and methods used in many studies. Here, indicate whether each material, system or method listed is relevant to your study. If you are not sure if a list item applies to your research, read the appropriate section before selecting a response.

Materials & experimental systems

n/a	Involved in the study	
☐	☒	Antibodies
☐	☒	Eukaryotic cell lines
☒	☐	Palaeontology and archaeology
☐	☒	Animals and other organisms
☒	☐	Human research participants
☒	☐	Clinical data
☒	☐	Dual use research of concern

Methods

n/a	Involved in the study	
☒	☐	ChIP-seq
☒	☐	Flow cytometry
☒	☐	MRI-based neuroimaging

Antibodies

Antibodies used	GAPDH: (14C10) Rabbit mAb #2118, lot:14 (Cell signaling)<br>p53:mixture of monoclonal antibodies DO1 + PAb1801 (home made prepared)<br>Goat anti-Rabbit IgG (H+L) Cross-Adsorbed Secondary Antibody, Alexa Fluor 647 anti-BrdU antibody: cat:A21244, lot:2872268 (Invitrogen)

Validation	GAPDH antibody was validated by the respective manufacturer (https://www.cellsignal.com/products/primary-antibodies/gapdh-14c10-rabbit-mab/2118).<br>DO1 and PAb1801 Anti-p53 Antibody is recommended for detection of wild type and mutant p53 of mouse, rat and human origin by WB, IP, and IF. Specificity of this antibody is also validated through use of our WT, null or mutant p53 cell lines (https://www.nature.com/articles/s41467-021-25359-z#additional-information, https://www.nature.com/articles/s41467-021-20928-8)<br>Alexa Fluor 647 anti-BrdU as validated by the respective manufacturer (https://www.thermofisher.com/antibody/product/Goat-anti-Rabbit-IgG-H-L-Cross-Adsorbed-Secondary-Antibody-Polyclonal/A-21244)

Eukaryotic cell lines

Policy information about cell lines

Cell line source(s)	SW480 were a kind gift from Varda Rotter lab (Commercial source:ATCC). RKO were a kind gift from Varda Rotter lab (Commercial source: American Type Culture Collection). HCT116 were obtained from ATCC. COLO205 were obtained from the NCI60 human tumor cell library in the Weizmann institute.
Authentication	SW480 cells were obtained from ATCC and were authenticated at the Ruth and Bruce Rappaport Faculty of Medicine using STR profiling. Other cell line were obtained from ATCC, NCI-60 and American Type Culture Collection and were not authenticated
Mycoplasma contamination	All cell lines tested negative for mycoplasma
Commonly misidentified lines<br>(See ICLAC register)	No commonly misidentified cell lines were used in this study.

Animals and other organisms

Policy information about studies involving animals; ARRIVE guidelines recommended for reporting animal research

Laboratory animals	Female 7 weeks old mice (C.B-17/IcrHsd-Prkdc-scid-Lyst-bg) were used in the study. All animals were housed in single-unit cages with 12-hr alternate light and dark cycles and at controlled ambient temperature (21-23 C) with humidity between 40%-60%
Wild animals	This study did not involve wild animals
Field-collected samples	This study did not involve samples collected from the field.
Ethics oversight	All mouse experiments were approved by the institutional animal care and use committee (IACUC) of the Weizmann Institute (approval 07200820-3).

Note that full information on the approval of the study protocol must also be provided in the manuscript.
